# Integrated multi-omics characterization across clinically relevant subgroups of long COVID

**DOI:** 10.1093/nsr/nwae410

**Published:** 2024-11-15

**Authors:** Jingwen Ai, Jingxin Guo, Ke Lin, Jianpeng Cai, Haocheng Zhang, Feng Zhu, Gangqiang Sun, Quanlin Xue, Kun Zhu, Yixuan Yang, Guanmin Yuan, Jieyu Song, Zhangfan Fu, Xiao Qi, Yuhan Sun, Weifang Lin, Chao Qiu, Ning Jiang, Sen Wang, Wenhong Zhang

**Affiliations:** Department of Infectious Diseases, Shanghai Key Laboratory of Infectious Diseases and Biosafety Emergency Response, National Medical Center for Infectious Diseases, Huashan Hospital, Shanghai Medical College, Fudan University, Shanghai 200040, China; Shanghai Sci-Tech InnoCenter for Infection and Immunity, Shanghai 20052, China; Department of Infectious Diseases, Shanghai Key Laboratory of Infectious Diseases and Biosafety Emergency Response, National Medical Center for Infectious Diseases, Huashan Hospital, Shanghai Medical College, Fudan University, Shanghai 200040, China; Shanghai Sci-Tech InnoCenter for Infection and Immunity, Shanghai 20052, China; Department of Infectious Diseases, Shanghai Key Laboratory of Infectious Diseases and Biosafety Emergency Response, National Medical Center for Infectious Diseases, Huashan Hospital, Shanghai Medical College, Fudan University, Shanghai 200040, China; Department of Infectious Diseases, Shanghai Key Laboratory of Infectious Diseases and Biosafety Emergency Response, National Medical Center for Infectious Diseases, Huashan Hospital, Shanghai Medical College, Fudan University, Shanghai 200040, China; Department of Infectious Diseases, Shanghai Key Laboratory of Infectious Diseases and Biosafety Emergency Response, National Medical Center for Infectious Diseases, Huashan Hospital, Shanghai Medical College, Fudan University, Shanghai 200040, China; Shanghai Sci-Tech InnoCenter for Infection and Immunity, Shanghai 20052, China; Department of Respiratory and Critical Care Medicine, Affiliated Wuxi Fifth Hospital of Jiangnan University, Wuxi Fifth People's Hospital, Wuxi 214000, China; Department of Infectious Diseases, Shanghai Key Laboratory of Infectious Diseases and Biosafety Emergency Response, National Medical Center for Infectious Diseases, Huashan Hospital, Shanghai Medical College, Fudan University, Shanghai 200040, China; Department of Infectious Diseases, Shanghai Key Laboratory of Infectious Diseases and Biosafety Emergency Response, National Medical Center for Infectious Diseases, Huashan Hospital, Shanghai Medical College, Fudan University, Shanghai 200040, China; Department of Infectious Diseases, Shanghai Key Laboratory of Infectious Diseases and Biosafety Emergency Response, National Medical Center for Infectious Diseases, Huashan Hospital, Shanghai Medical College, Fudan University, Shanghai 200040, China; Department of Infectious Diseases, Shanghai Key Laboratory of Infectious Diseases and Biosafety Emergency Response, National Medical Center for Infectious Diseases, Huashan Hospital, Shanghai Medical College, Fudan University, Shanghai 200040, China; Department of Infectious Diseases, Shanghai Key Laboratory of Infectious Diseases and Biosafety Emergency Response, National Medical Center for Infectious Diseases, Huashan Hospital, Shanghai Medical College, Fudan University, Shanghai 200040, China; Department of Infectious Diseases, Shanghai Key Laboratory of Infectious Diseases and Biosafety Emergency Response, National Medical Center for Infectious Diseases, Huashan Hospital, Shanghai Medical College, Fudan University, Shanghai 200040, China; Department of Infectious Diseases, Shanghai Key Laboratory of Infectious Diseases and Biosafety Emergency Response, National Medical Center for Infectious Diseases, Huashan Hospital, Shanghai Medical College, Fudan University, Shanghai 200040, China; Department of Infectious Diseases, Shanghai Key Laboratory of Infectious Diseases and Biosafety Emergency Response, National Medical Center for Infectious Diseases, Huashan Hospital, Shanghai Medical College, Fudan University, Shanghai 200040, China; Department of Infectious Diseases, Shanghai Key Laboratory of Infectious Diseases and Biosafety Emergency Response, National Medical Center for Infectious Diseases, Huashan Hospital, Shanghai Medical College, Fudan University, Shanghai 200040, China; Department of Infectious Diseases, Shanghai Key Laboratory of Infectious Diseases and Biosafety Emergency Response, National Medical Center for Infectious Diseases, Huashan Hospital, Shanghai Medical College, Fudan University, Shanghai 200040, China; Shanghai Sci-Tech InnoCenter for Infection and Immunity, Shanghai 20052, China; Department of Infectious Diseases, Shanghai Key Laboratory of Infectious Diseases and Biosafety Emergency Response, National Medical Center for Infectious Diseases, Huashan Hospital, Shanghai Medical College, Fudan University, Shanghai 200040, China; Shanghai Sci-Tech InnoCenter for Infection and Immunity, Shanghai 20052, China; Department of Infectious Diseases, Shanghai Key Laboratory of Infectious Diseases and Biosafety Emergency Response, National Medical Center for Infectious Diseases, Huashan Hospital, Shanghai Medical College, Fudan University, Shanghai 200040, China; Shanghai Sci-Tech InnoCenter for Infection and Immunity, Shanghai 20052, China; Department of Infectious Diseases, Shanghai Key Laboratory of Infectious Diseases and Biosafety Emergency Response, National Medical Center for Infectious Diseases, Huashan Hospital, Shanghai Medical College, Fudan University, Shanghai 200040, China; Shanghai Sci-Tech InnoCenter for Infection and Immunity, Shanghai 20052, China; Institute of Infection and Health, Fudan University, Shanghai 200040, China

**Keywords:** post-acute sequelae of COVID-19, long COVID subgroups, proteogenomic features, heterogeneity

## Abstract

When SARS-CoV-2 became regional epidemics, a substantial number of patients suffered from post-acute sequelae of COVID-19 (PASC, aka long COVID). Exploring the pathogenesis and especially the heterogenicity features of long COVID subgroups is of paramount importance for understanding its etiology. In this study, through integrative multi-omics analyses encompassing transcriptomics, proteomics, and metabolomics, long COVID patients exhibited overall elevated MAPK pathway activation, while patients who have recovered from long COVID showed down-regulation of this response. Long COVID heterogenicity is described by multi-omics distinct signatures for each subgroup. The Multisystemic (MULTI) symptom subgroup is characterized by enhanced glycerophospholipid and ether lipid metabolism, Neurological (NEU) by augmented glycoprotein synthesis metabolism, Cardio cerebral (CACRB) by increased pyruvate metabolism and suppressed macrophage polarization, Musculoskeletal + Systemic (MSK + SYST) by elevated glycerophospholipid metabolism, and Cardiopulmonary (CAPM) by inhibited NF-κB signaling pathways. ABHD17A, CSNK1D, PSME4 and SYVN1 were general long COVID combination biomarkers, while CRH (MULTI), FPGT (NEU), CBX6 (CACRB) and RBBP4 (CAPM) were selected as serum-specific subgroup proteins. Our study provides a commonly shared and distinct pathophysiological explanation underpinning PASC, paving the way for future diagnosis and therapeutic interventions.

## INTRODUCTION

Since the onset of 2020, a plethora of global research endeavors have documented the manifestation of post-acute sequelae of SARS-CoV-2 infection (PASC), commonly known as long COVID, in a subset of patients, with prevalence rates ranging from 1.9% to 20% [1–4]. Despite ongoing investigations, the long-term implications of PASC on populations in the post-pandemic era remain insufficiently elucidated. A pivotal factor contributing to this knowledge gap is the pronounced heterogeneity of long COVID clinical symptomatology across individuals. For instance, a segment of patients presents systemic symptoms epitomized by fatigue, whereas others report cardiovascular manifestations, including palpitations and chest pain. Notably, the phenomenon of ‘brain fog’, characterized by insomnia and diminished memory, has garnered significant attention [5]. Symptoms of long COVID as reported globally appear to span virtually all physiological systems, encompassing cardiovascular, gastroenterological, musculoskeletal, and respiratory domains. This raises critical inquiries regarding which long COVID symptoms may precipitate more severe disease burdens and enduring impacts. Furthermore, it posits the question of whether the pathophysiological mechanisms underlying long COVID diverge based on the symptomatically affected system. Investigating the multi-omics and immune response variances among patients exhibiting specific long COVID symptoms would yield pivotal insights into the aforementioned quandaries.

Various investigations have delineated the immunopathological underpinnings of patients suffering from long COVID, leveraging omics and immunological methodologies. Similar to the pronounced heterogeneity observed in clinical manifestations, discrepancies in immunological profiles among long COVID patients have been documented across different studies. Notably, certain researches have highlighted aberrant activation within T cell pathways among individuals [6,7], whereas others have identified enhanced activity in pathways associated with neutrophils or the humoral immune response [8–10]. Moreover, distinct immunological subtypes within long COVID patient populations have been uncovered, potentially correlating with divergent clinical characteristics and prognoses [11]. Despite attempts by some studies to pinpoint immune signatures associated with specific clinical phenotypes [12], a comprehensive, systematic analysis elucidating the relationship between the immune homogeneity, heterogeneity and various clinical symptomatology subgroups within long COVID remains absent.

To address the aforementioned questions, since 2022, we have established a cohort of over 20 000 individuals in Shanghai infected with the Omicron variant and conducted a year-long follow-up, during which we dynamically assessed the incidence of long COVID symptoms and their sub-group characteristics. Concurrently, serial blood samples from different clinical symptomatic sub-groups were collected and subjected to multi-omics analyses to explore their specific immunological patterns and prognostic markers. Furthermore, as dynamic zero-COVID policy was implemented in the mainland of China from 2020 to 2022, our cohort was shielded from biases that could arise from repeated infections with different strains or reinfections with the Omicron variant. In the context of a global confirmed COVID-19 infection rate surpassing 10% and high number of individuals reporting long COVID symptoms, the findings of this study will provide critical data for exploring the heterogeneity in clinical and immunological characteristics of long COVID patients, as well as their clinical outcomes. This research will also significantly contribute to the precision diagnosis and treatment decisions for long COVID patients in the future.

## RESULTS

### Clinical classification and characteristics of long COVID

**Figure 1.**
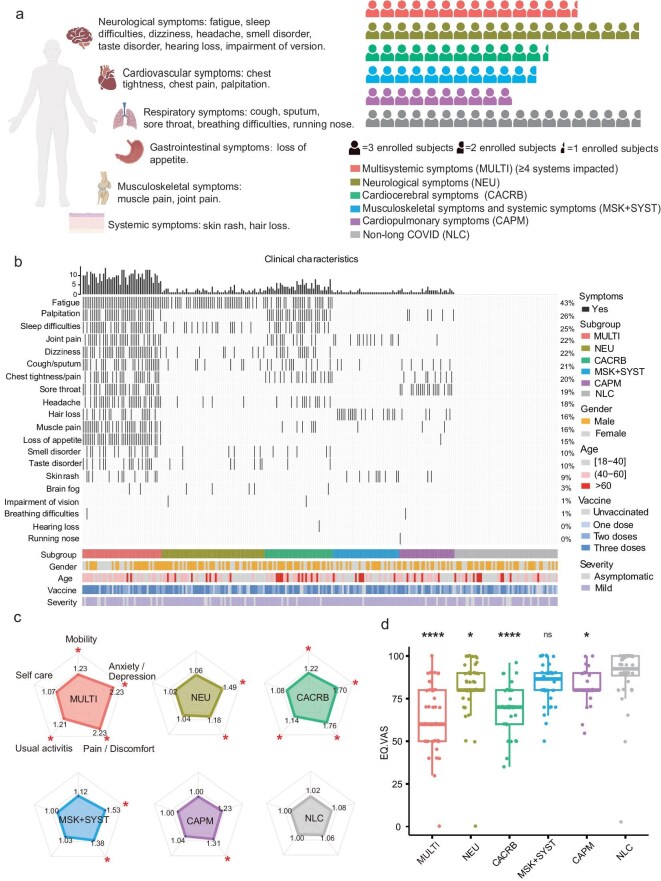
Clinical heterogeneity of long COVID. (a) Overview of clinical subgroups within long COVID patients. (b) Symptomatology and related clinical characteristics of the 255 participants. (c) Radar charts of the EQ-5D-5 L scale for the six clinical subgroups. (d) Box plots of the EQ-VAS scores for the six clinical subgroups.

Among the 201 long COVID patients enrolled in this investigation, the most commonly reported symptoms were fatigue (43.1%), palpitations (26.4%), and sleep disturbances (25.2%). Based on our predefined classification criteria listed in the Methods, the study finally included 43 patients in the Multisystemic (MULTI) group (≥4 systems impacted), 56 in the Neurological (NEU) group, 37 in the Cardio cerebral (CACRB) group, 35 in the Musculoskeletal + Systemic (MSK + SYST) group, 30 in the Cardiopulmonary (CAPM) group, and 56 in the Non-long COVID (NLC) group (Fig. 1a, b, Table S1). Of these patients, 241 (93.8%) completed the EQ-5D-5 L questionnaire and EQ-VAS assessment. Within the EQ-5D-5 L domain scores, the MULTI group reported significantly greater difficulties in mobility (1.23 vs 1.02, *P* = 0.007), performing usual activities (1.21 vs 1.00, *P* = 0.007), experiencing pain/discomfort (2.23 vs 1.06, *P* < 0.001), and anxiety/depression (2.23 vs 1.08, *P* < 0.001) compared to the NLC group; the NEU group reported notably higher levels of pain/discomfort (1.18 vs 1.06, *P* = 0.003) and anxiety/depression (1.49 vs 1.08, *P* < 0.001); the CACRB group indicated significant challenges with self-care (1.08 vs 1.00, *P* = 0.043), conducting usual activities (1.14 vs 1, *P* = 0.008), pain/discomfort (1.76 vs 1.06, *P* < 0.001), and anxiety/depression (1.70 vs 1.08, *P* < 0.001); the MSK + SYST group reported a significant increase in pain/discomfort (1.38 vs 1.06, *P* = 0.001) and anxiety/depression (1.53 vs 1.08, *P* < 0.001); and the CAPM group reported a notably higher extent of pain/discomfort (1.31 vs 1.06, *P* = 0.002) compared to the NLC group (Fig. 1c). Regarding the EQ-VAS scores, the MULTI group (64.8 vs 89.7, *P* < 0.001), CAPM group (83.4 vs 89.7, *P* = 0.048), CACRB group (68.6 vs 89.7, *P* < 0.001), and NEU group (81.6 vs 89.7, *P* = 0.014) all reported significantly reduced average self-reported health status as compared to the NLC group; the MSK + SYST group did not show a significant difference (84.5 vs 89.7, *P* = 0.077) (Fig. 1d).

We evaluated the symptom relief rates across the subgroups at 12-month post COVID-19 infection. Out of the 257 subjects, 99 participated in the 12-month follow-up assessment, with 17 individuals self-reporting instances of reinfection having been ruled out from further study. Within the cohort of 82 patients without reported reinfection, 13 belonged to the MULTI group, 19 to the NEU group, 16 to the CACRB group, 19 to the MSK + SYST group, and 15 to the CAPM group. At the 12-month evaluation, the MULTI group exhibited complete symptom relief in 69.2% (9/13) of patients and partial relief in 30.8% (4/13); the NEU group showed complete relief in 79.0% (15/19), partial relief in 10.5% (2/19), and non-relief in 10.5% (2/19); the CACRB group had complete relief in 75.0% (12/16) and partial relief in 25.0% (4/16); the MSK + SYST group reported complete relief in 94.7% (18/19) and non-relief in 5.3% (1/19); and the CAPM group demonstrated complete relief in 93.3% (14/15) and partial relief in 6.7% (1/15).

### Multi-omics analysis between long and non-long COVID

**Figure 2.**
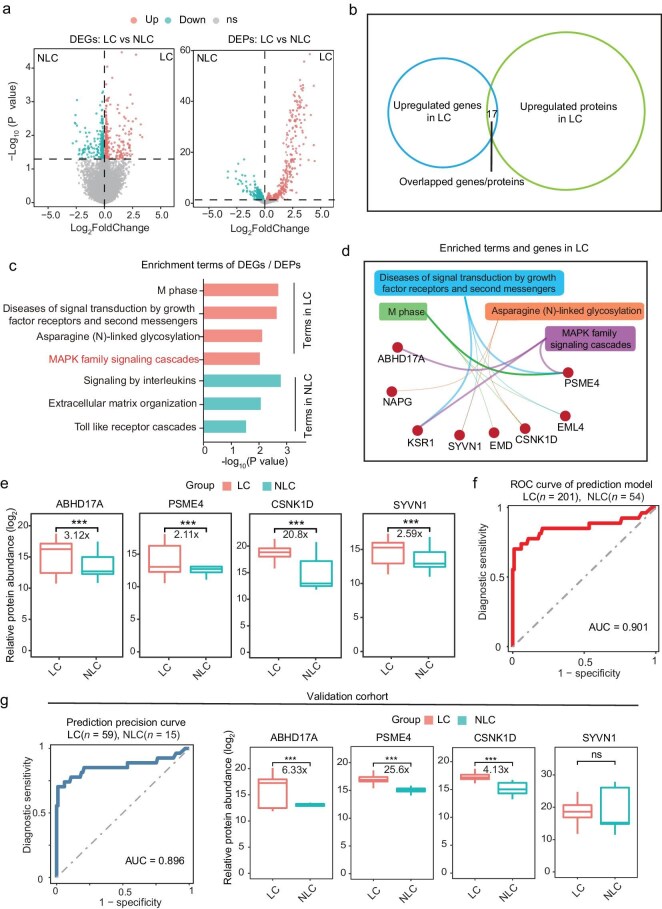
Proteogenomic landscape of long COVID and non-long COVID. (a) Volcano plots of differential analysis of expression genes (right) and proteins (left) between long COVID (LC) and non-long COVID (NLC) groups. Red indicates genes/proteins significantly upregulated in the long COVID group, green indicates genes/proteins significantly downregulated in the long COVID group, and gray indicates genes/proteins with no significant difference in expression. (b) Venn plot showing the overlapped genes and proteins upregulated in LC. (c) Bar plot of pathway enrichment analysis showing significant differences at both gene and protein levels between long COVID and non-long COVID groups. Red indicates pathways significantly upregulated in the long COVID group, and green indicates pathways significantly downregulated in the long COVID group, *P* value <0.05. (d) Protein-Protein interaction networks of proteins and pathways upregulated in long COVID. (e) Box plots showing proteins with significant differences in panel (d). Red indicates the long COVID group, and green indicates the non-long COVID group. (f) ROC curve for the diagnosis model to identify long COVID and non-long COVID groups, using the protein combination in panel (e). (g) ROC curve showing the precision of the diagnosis model and relative protein abundance between long COVID and non-long COVID group in the validation cohort.

Transcriptomic analyses were performed on peripheral whole blood and comparative proteomic assessments were conducted on plasma from long COVID and non-long COVID individuals. After quality control and filtering, 201 LC samples and 54 NLC samples were finally analyzed. The analytical approach encompassed differential gene and protein expression profiling, which elucidated proteins exhibiting significant differential expression at both the transcript and protein levels (Fig. 2a, b). Subsequent enrichment analyses delineated markedly divergent biomolecular signatures between the two cohorts. The long COVID group exhibited distinct enrichment in MAPK family signaling cascades, asparagine (N)-linked glycosylation, M phase and cell signal transduction pathways (Fig. 2c), and a significant elevation of several associated proteins was observed, including ABHD17A [13,14] and PSME4 [15], which are implicated in the MAPK signaling cascade; CSNK1D [16, 17], SYVN1 [18,19], indicating activation in cell cycling and protein modification processes [20] (Fig. 2d, e). Notably, these four proteins combined together possessed the best robust discriminative diagnostic potential for differentiating between long COVID and non-long COVID states (AUC = 0.901) (Fig. 2f, Fig. S1A). We also validated these four biomarkers in an external validation cohort (59 LC and 15 NLC samples), and the diagnostic efficacy reached an AUC of 0.896 as well as with a combination of these markers (Fig. 2g).

We also found other proteins, such as EMD, STX4, etc., that showed statistic significances between LC and NLC groups, but had relatively smaller average differences (Fig. S1B). Furthermore, metabolomic evaluations revealed an upregulation of several metabolism pathways in the long COVID group (Fig. S2A–C). Therefore, further studies might be required to validate their biological differences.

### MAPK family signaling activation in long COVID symptoms persistence

**Figure 3.**
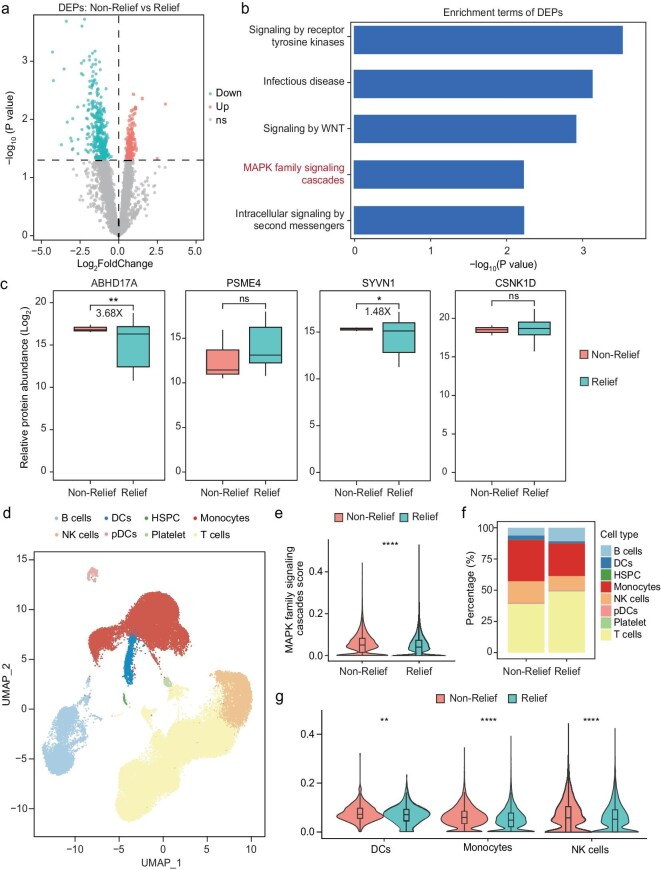
MAPK family signaling activation in long COVID symptoms persistent groups. (a) Volcano plot of differential proteins between Relief and Non-Relief groups. Red indicates proteins significantly upregulated in the Relief group, green indicates proteins significantly downregulated in the Relief group, and gray indicates proteins with no significant difference in expression. (b) Enrichment pathway analysis of upregulated proteins in the Relief group. (c) Box plots of four diagnostic proteins between the Relief and Non-Relief groups. Red represents the Relief group, and green represents the Non-Relief group. (d) UMAP plot of single-cell RNA sequencing data from the validation cohort, showing peripheral blood mononuclear cells (PBMCs) from all participants. (e) Violin plot illustrating the MAPK Family Signaling Cascades gene score between the Relief and Non-Relief groups (all cells). (f) The distribution of cell clusters in the Relief and Non-Relief groups. (g) Violin plot illustrating the MAPK Family Signaling Cascades gene score within DCs, monocytes and NK cells between the Relief and Non-Relief groups.

To seek potential pathways that relate to the persistence of long COVID symptoms, 82 patients without reinfections who agreed to the one-year follow-up were categorized into a Relief cohort (*n* = 79) and Non-Relief cohort (*n* = 3), based on whether symptoms persisted at the 12-month follow up post-infection. A differential proteomic analysis conducted at 6-months post infection (Fig. 3a) showed enriched MAPK family signaling cascades pathways in Non-Relief cohorts (Fig. 3b). We further tested the prognostic potential of ABHD17A, PSME4, CSNK1D, and SYVN1, which had showed diagnostic ability between LC and NLC groups, and the result showed that ABHD17A had a 3.68-fold increase in the 12-month Non-Relief cohort (Fig. 3c). To validate the aforementioned results via cellular approaches, we performed single cell sequencing in residual samples among subjects in 1-year Relief and Non-Relief groups. We procured peripheral blood mononuclear cells (PBMCs) from these individuals at the 6-month post-infection for single-cell RNA sequencing, and the PBMCs underwent dimensional reduction and were classified into 8 discrete cellular subpopulations based on their transcriptomic characteristics (Fig. 3d). An upregulation of MAPK family signaling scoring was also observed in the Non-Relief cohort (Fig. 3e), consistent with previous proteomic data insights. This comparative analysis also elucidated the differential representation of cellular subpopulations between the two cohorts and showed that the Non-Relief cohort had a higher percentage of monocyte and NK cells distribution (Fig. 3f). Further analysis found that MAPK family signaling scoring in DCs, monocytes, and NK cells shared an elevation trend in the Non-Relief group, compared to the Relief group (Fig. 3g).

We further analyzed other proteomic signatures that were distinct between the 1-year Relief and Non-Relief cohorts (Fig. S3A). The serum of Relief cohort patients was enriched with proteins involved in immune responses and vesicle-mediated transport, such as CDH1, PRKAG2, and LGALS3BP, etc.; conversely, the Non-Relief cohort exhibited an abundance of proteins such as DDOST, associated with asparagine N-linked glycosylation, and LAMTOR2 and PRDX5, which participate in TP53-regulated metabolic gene pathways. Employing random forest modeling to accurately identify individuals with potential for symptom resolution 12 months post-infection, it was found that some proteins possessed commendable predictive utility for PASC outcomes with an average Receiver Operating Characteristic (ROC) Area Under Curve (AUC) exceeding 0.8, as determined across 100 model iterations featuring randomized training/testing dataset divisions (Fig. S3B). However, further animal model studies might be required to validate their biological differences.

### Specific immune characteristics of clinical subgroups of long COVID

**Figure 4.**
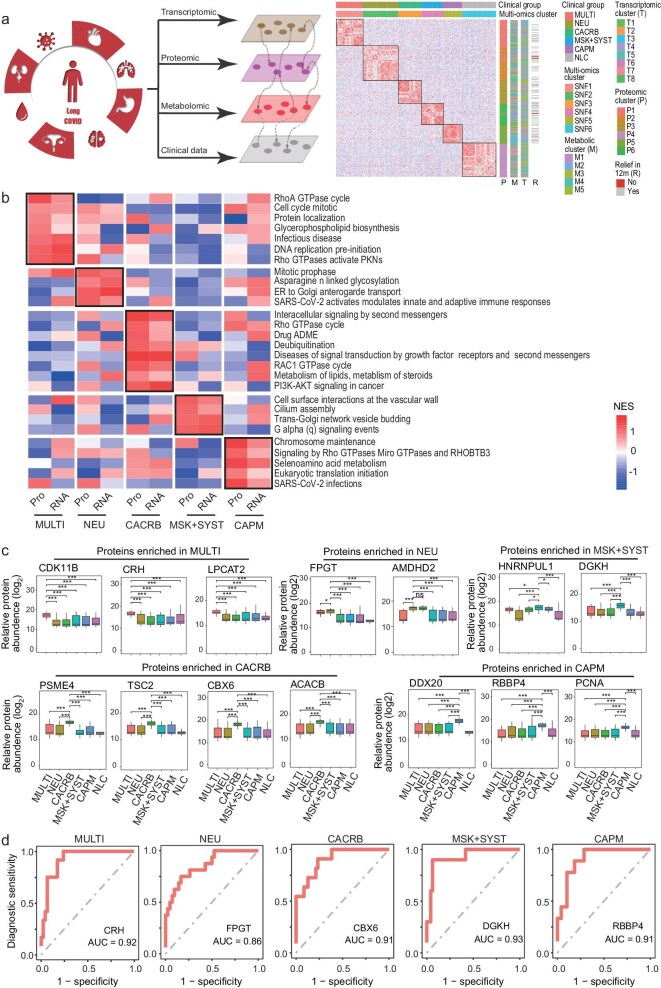
Heterogeneity of long COVID subgroups. (a) Schematic overview of multi-omics classification. (b) Integrated analysis of altered pathways at transcriptome and proteome levels among the six clinical subgroups. (c) Relative abundance of main differential proteins among clinical subgroups (*t*-test). Boxes indicate interquartile range (IQR) with a line at the median. Whiskers represent the minimum and maximum values at most 1.5* IQR from the quartiles. (d) ROC curves for using our MNN models to identify potential protein biomarkers to differentiate between long COVID clinical subgroups.

To elucidate the underlying biological signatures of long COVID symptoms within distinct clinical subtypes, a multi-omics clustering approach was adopted utilizing Similarity Network Fusion (SNF) across proteomic, transcriptomic, and metabolomic datasets. The proteomic clustering demonstrated a high concordance with the defined clinical subtype phenotypes (Fig. 4a, Fig. S4A, Table S2), indicating that patients with long COVID exhibit proteomic profiles that are distinct to their clinical manifestations. In the multisystem impacted group (MULTI), there was an upregulation of proteins related to stress response (CRH) [21,22], cell cycle modulation (CDK11B) [23–25], and glycerophospholipid/ether lipid metabolism (LPCAT2) [26]. The neurological systems group (NEU) was characterized by elevated proteins involved in glucometabolic, such as FPGT and AMDHD2, which are also implicated in glycoprotein regulation [27–30]. The cardio cerebral symptom group (CACRB) presented increased levels of PSME4, which is associated with MAPK family signaling cascades, and this finding was similar to what we had observed in the LC group. Besides, we also found increased proteins associated with macrophage suppression, such as TSC2 [31], CBX6 [32], and protein ACACB, which is involved in pyruvate metabolism and fatty acid synthesis [33]. The cardiopulmonary subgroup (CAPM) exhibited an increase in DDX20 [34,35] which was involved in downregulating the NF-κB signaling pathway, whereas the musculoskeletal + systemic group (MSK + SYST) showed an elevation in proteins facilitating glycerophospholipid metabolism, such as DGKH [36,37] (Fig. 4b, c). Signature proteins were selected from each clinical subgroup, CRH for the MULTI group, FPGT for the NEU group, CBX6 for the CACRB group, DGKH for the MSK + SYST group, and RBBP4 for the CAPM group, and a Convolutional Neural Network (CNN) diagnostic model was developed through successive training iterations. The resulting diagnostic efficacy, as measured by the area under the receiver operating characteristic curve (AUC), was 0.92 for the MULTI group, 0.86 for the NEU group, 0.91 for the CACRB group, 0.93 for the MSK + SYST group, and 0.91 for the CAPM group (Fig. 4d, Fig. S4B, and Fig. S5).

The validation cohort also showed the same trends, with CRH exhibiting a higher expression in the MULTI group, FPGT in the NEU group, CBX6 in the CACRB group, and RBBP4 in the CAPM group. Additionally, they all showed higher expression compared to the NLC group (Fig. S6A).

**Figure 5.**
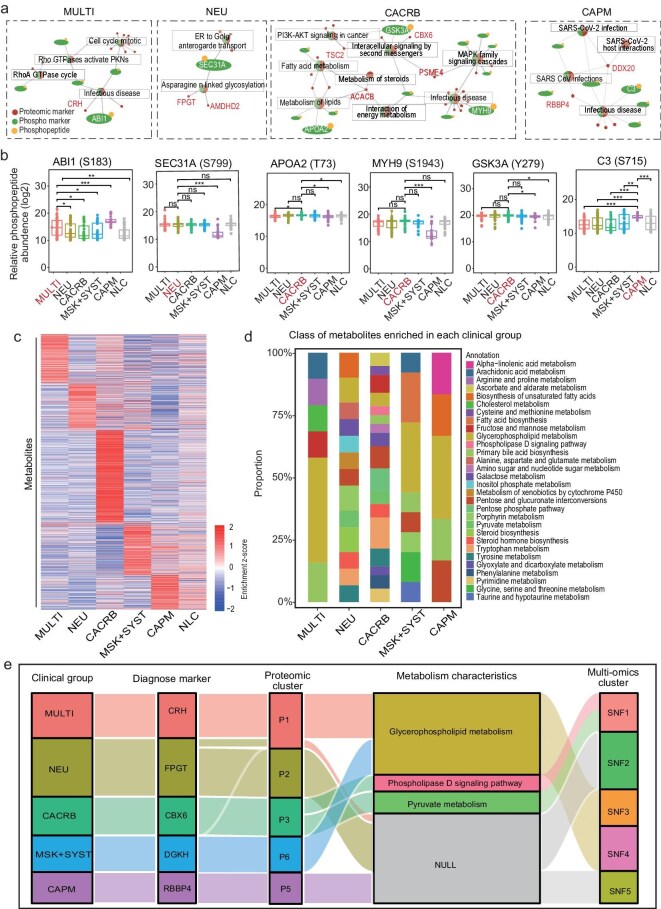
Metabolomic profiles of six clinical subgroups. (a) Overview of the targeted proteins and phosphorylation of proteins which are closely associated with them, enriched in the same pathways, among clinical subgroups. (b) Boxplots show the different enrichment levels of protein phosphosites among clinical subgroups. (c) Heatmap showing the enriched metabolites heterogeneity within each clinical subgroup. (d) Classification of metabolites enriched in each clinical subgroup. (e) Sankey diagram depicting the result of integrative multi-omics analysis, showing the flow of diagnosed protein analysis, proteome cluster assignment, metabolic characteristics and multi-omics classification of long COVID.

To further understand the activity of targeted proteins, we explored the significantly altered phosphosites involved in enriched pathways of each clinical subtype (Fig. 5a, b, Fig. S6B). In the MULTI group, we identified activation of CRH-related infectious disease response pathways, marked by ABL1 phosphorylation at S183. ABL1 is known to be essential for the induction of pro-inflammatory cytokines [38,39]. In the NEU group, we identified activation of FPGT and AMDHD2 related glycosylation response pathways, marked by SEC31A phosphorylation at S799, which could join glycosylation and thus may promote inflammatory responses [40,41]. In the CACRB group, we identified activation of fatty acid synthesis protein ACACB which was related to lipid metabolism, marked by Apolipoprotein A2 (APOA2) phosphorylation at T73. We also identified activation of the PSME4-related infectious diseases response pathway, marked by MYH9 phosphorylation at S1943. MYH9 was identified as an ACE2 coreceptor to promote SARS-CoV-2 infection of human lung cells [42]. What's more, we identified activation of CBX6-related intracellular signaling by second messengers, marked by GSK3A phosphorylation at Y279, which is a regulator of the inflammatory response [43,44]. In the CAPM group, we identified activation of DDX20- and RBBP4-related infectious disease response pathways, marked by C3 phosphorylation at S715, which is a well-known pro-inflammatory molecule [45,46].

### Specific metabolic characteristics of clinical subgroups of long COVID

In the ensuing analysis, we delineated the metabolomic disparities across the five clinical subtypes. We found that metabolite enrichment showed diversity among all 5 clinical groups (Fig. 5c, d, Fig. S7). In consistence with proteomic clusters, the MULTI and MSK + SYST subgroups were characterized by an upregulation of glycerophospholipid metabolism, and the CACRB subgroup was typified by augmented phospholipase D signaling pathway and pyruvate metabolism. We produced a Sankey diagram, depicting that these metabolic traits were in concordance with the proteomic patterns previously observed (Fig. 5e).

## DISCUSSION

In this study, we reported a large-scale proteogenomic profile of overall and different clinical subtypes of long COVID, involving transcriptomics, proteomics, metabolomics, and phosphoproteomics analyses. These data may provide a valuable resource for further unveiling the immune features underneath the clinical homogeneous and heterogeneous manifestation and facilitate potential subgroup-specific immunotherapy.

Globally, long COVID symptoms are characterized by significant clinical and immune heterogenicity among populations. In our study, all long COVID patients, regardless of the subgroups, exhibited elevated MAPK activation, and patients with stronger MAPK activation showed longer persistence of the symptoms up till 12 months. Although consensus has been reached that immune dysregulation is the one of the main potential causes of long COVID symptoms [47], few studies have focused on the commonly shared immune signatures within different long COVID subgroups. Enrichment of MAPK activation had been previously reported in fatigue dominant long COVID patients [48], and the SARS-CoV-2 S1 spike protein could promote and trigger the MAPK signaling pathway [49]. Our results indicated that MAPK signaling pathway might be an important immune target in the therapeutic approach to long COVID.

**Figure 6.**
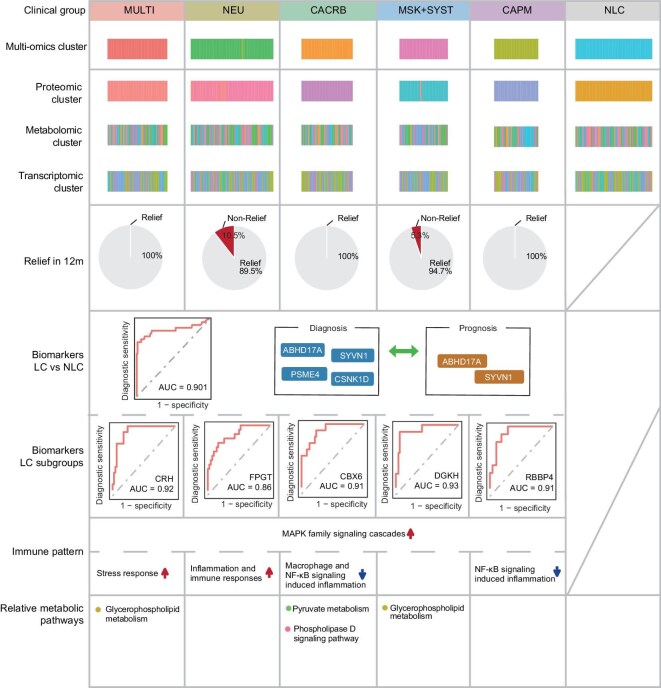
Graphical summary showing the major clinical and immune features of 201 long COVID patients. Heatmap showing unbiased consensus clustering of proteomic clusters, transcriptomic clusters and metabolomic clusters; pie charts representing symptom relief rate; novel biomarkers and their ROC curves; immune pattern and relative metabolic pathways.

Our study further identified 5 unique clinical subgroups of long COVID through multi-omics classification with the combined transcriptomics, proteomics, and phosphoproteomics data, which was consistent with clinical subtypes (Fig. 6). Patients with multisystemic and musculoskeletal (MULTI, MSK + SYST) symptoms had significantly increased glycerophospholipid and ether lipid metabolisms, which might partially explain the myalgia expressed by some participants. Patients with post-COVID neurological symptoms (NEU) had increased glycoprotein biological activity and carbohydrate metabolism, which could mediate inflammation and immune responses [4]. Such findings are in accordance with numerous studies that have reported increased risk of incident neurologic sequelae including episodic disorders and encephalitis [50,51]. Patients with both cardiovascular + neurological symptoms (CACRB) showed pyruvate metabolism and elevated fatty acid synthesis [52], which relate closely with the risk of arterial and venous thrombotic events post-COVID [53,54]. What's more, our study further revealed different expression patterns of immune responses. Patients in the MULTI group showed elevated stress responses, while CACRB and CAPM groups exhibited inhibition of the macrophage and NF-κB signaling pathway, respectively. These data correlated with previous studies that showed immunity-metabolism imbalance might contribute to the development of long COVID [55,56]. But as the heterogenous features among 5 subgroups in our study has showed, one important key to understanding the immune patterns of different subtypes of long COVID patients might lie in their distinct clinical manifestations. Our study further found that MSK + SYST and NEU groups had a lower 1-year relieve rate than the others, suggesting that patients with these symptoms might endure a longer recovery period. This finding was also in accordance with previous published data in which researchers found different recovery modes of biological processes [57]. However, although multiple studies have published several multi-omics data concerning the heterogenicity in biological processes and recovery mode, one obstacle hindering further mechanism study has been the lack of a long COVID animal model. Recently, a study has successfully set up a mouse model of lung PSAC, which might remarkably benefit future exploration of potential therapeutic approaches [58,59].

Among 14 proteins that we found could differentiate between different clinical long COVID groups, we further identified CRH (MULTI), FPGT (NEU), CBX6 (CACRB), DGKH (MSK + SYST), and RBBP4 (CAPM), as putative biomarkers. With CSNK1D, ABHD17A, PSME4, and SYVN1 to differentiate between long COVID and non-long COVID, these combinations of 9 biomarkers could effectively facilitate the accurate identification of patients suffering from specific long-COVID symptoms. Among which, CRH is closely related with increased carbohydrate and lipid metabolism, and peripheral CRH can directly exert proinflammatory effects [60]. FPGT encodes fucose-1-phosphate guanylyltransferase, a protein crucial for fucose metabolism, which plays a significant role in neuronal development [61,62]. Patients in the MSK + SYST group had elevated DGKH, which could cause the muscle pain as DGKH participates in the body's phospholipase signaling, what's more, study has shown that DGKH expression may limit the DAG-induced antiviral immune response as negative feedback during SARS-CoV-2 infections [63,64]. The role of CDK11B is closely related to increased glycerophospholipid metabolism, while AMDHD2 has been reported through previous study to mediate immune system dysfunction and increase the risk of cancer, whether it could lead to neurological immune dysfunction during or post COVID is therefore worth further study [29,65–67]. Apart from being known to be actively involved in the body's fatty acid synthesis, aberrant expression of the ACACB genes could also raise the risk for cancer.

Our study has the following limitations. First, the current conclusion is based on retrospective data analysis and further prospective trials including a larger number of subjects should be conducted to provide robust evidence, especially including more aged populations. Second, for the proteomic and metabolic phenotype observed among different long COVID subgroups, more experimental or animal model research should be done for further validation. Furthermore, this retrospective study was conducted among a homogeneous population in China and a further validation cohort among heterogeneous populations might further benefit our understanding of the disease.

In summary, our study, based on a large-scale multi-omics cohort, comprehensively profiled the homogeneity and heterogeneity of long COVID from clinical and integrated transcriptomic, proteomic, and metabolic characteristics, revealing the underlying common and unique molecular and immune mechanisms for each long COVID subgroup that were not fully captured by clinical manifestation analysis. The information provided by this study may open new paths for the development of personalized individualized therapy to eventually benefit clinical practice. We hope that the observations and analysis described here, which indicated shared and individualized immune characteristics among patients, will be a rich resource for the in-depth study of the pathogenesis, progression, and therapeutics of long COVID.

## METHODS

### Study design

This investigation encompassed 255 subjects who were admitted to Huashan Hospital of Fudan University, Shanghai, for COVID-19 treatment during the prevalence of the Omicron BA.2 variant in 2022, all of whom had documented positive tests for SARS-CoV-2 nucleic acid or antigen. The criteria for inclusion of PASC patients, along with the associated instruments (Long COVID symptom inventory, EQ-5D-5 L, GAD-7, PHQ-9, and PTSD questionnaires), have been delineated in our prior studies regarding this cohort [68]. A longitudinal follow-up of these patients was conducted over a span of 1 year. Within the EQ-5D-5 L questionnaire, a scoring metric was employed, ranging from 1 (indicating no issues) to 4 (indicating extreme issues), to quantitatively assess the extent of difficulties encountered by the participants across various dimensions. At the time of follow-up, peripheral blood samples were procured from the patients for analyses including transcriptomics, proteomics, and metabolomics. And they also underwent clinical examinations such as CT scans and routine blood tests, detailed in Table S1.

Drawing upon prior research, symptoms of long COVID such as throat discomfort, nasal congestion, cough with expectoration, and respiratory distress were categorized under respiratory system manifestations; sensations of chest tightness and palpitations were ascribed to cardiovascular system manifestations; symptoms including fatigue, disturbances in sleep, dizziness, diminished visual acuity, headache, dysgeusia, anosmia, and cognitive fog were grouped under neurological manifestations; myalgia and arthralgia were identified as musculoskeletal system manifestations; alopecia and dermatologic rash were regarded as systemic manifestations; and reduced appetite was classified under digestive system manifestations. Subjects presenting concomitantly with respiratory and cardiovascular symptoms were allocated to the Cardiopulmonary (CAPM) cluster; those exhibiting mainly both respiratory and neurological symptoms were allocated to the Cardio cerebral (CACRB) cluster; individuals with exclusively neurological symptoms were assigned to the Neurological symptoms (NEU) cluster; those manifesting both musculoskeletal and systemic symptoms were assigned to the Musculoskeletal symptoms + Systemic (MSK + SYST) cluster; participants displaying symptoms across four or more systems were categorized into the Multisystemic (MULTI) cluster; and those devoid of long COVID symptoms were classified into the Non-long COVID (NLC) cluster.

We also set up a validation cohort to test our result, and recruited 59 long COVID patients, including 21 in the MULTI group, 10 in the NEU group, 6 in the CACRB group, 9 in the MSK + SYST group, and 13 in the CAPM group. Additionally, we recruited 15 post–COVID-19 patients without long COVID (NLC group) to serve as an independent external validation cohort alongside the long COVID patients.

### Ethics statement

All participants signed written informed consent forms to participate in the baseline and follow-up surveys. Informed consent was obtained from the study participants before completing the study questionnaire. The study was approved by the Ethics Committee of Huashan Hospital, Shanghai, China (2023-750).

## Supplementary Material

nwae410_Supplemental_Files

## Data Availability

Processed data is listed in Tables S3–S7. Raw data were deposited in the National Genomics Data Center: HRA007867. The codes are available from the corresponding authors upon reasonable request.
