# Supplementary material for: Integrated multi-omics characterization across clinically relevant subgroups of long COVID
Source: Natl Sci Rev. 2024 Nov 15;12(8):nwae410. doi: 10.1093/nsr/nwae410 (PMC12365753; doi:10.1093/nsr/nwae410)
Supplement: nwae410_Supplemental_Files [file nwae410_supplemental_files.zip › Supplementary document.pdf]

Supplementary Materials

Contents:

Supplementary Figure 1: Proteomic feature of long COVID and non-long COVID

A. ROC curve for Diagnostic ability of ABHD17A，PSME4，CSNK1D and SYVN1 to identify long COVID and non-long COVID group.

B. Box plots shows other potential proteins with significant differences between long COVID and non-long COVID group. Red indicates the long COVID group, green indicates the non-long COVID group.

Supplementary Figure 2: Metabolomic feature of long COVID and non-long COVID

A. Volcano plot of differential metabolite analysis between long COVID and non-long COVID groups. Red indicates metabolites significantly upregulated in the long COVID group, green indicates metabolites significantly downregulated in the long COVID group, and gray indicates metabolites with no significant difference in expression.

B. Bubble plots shows pathway enrichment analysis of differential metabolites. The left represents pathways enriched by upregulated metabolites in the long COVID group; the right represents pathways enriched by downregulated metabolites in the long COVID group.

C. Heatmaps of metabolomic signatures between long COVID and non-long COVID groups.

Supplementary Figure 3: Potential prognostic proteins and analysis of their relative protein abundance between the Relief and Non-Relief groups

A. Box plot of differential proteins between the Relief and Non-Relief groups. Red represents the Non-Relief group, and green represents the Relief group.

B. ROC curve analysis for potential protein’s ability to predict symptom resolution 12 months post-infection using random forest modeling

Supplementary Figure 4: Homogeneity evaluation of multi-omics clustering results and ROC curves analysis of pathway enrichment for powerful evaluation in each clinical subgroup

A. Consistency between clinical subgroups and multi-omic clustering outcome.

B. ROC curves analysis of pathway enrichment to identify each clinical subgroup.

Supplementary Figure 5: Violin plot illustrating the gene set expression level of selected terms in each clinical subgroup

Supplementary Figure 6. Relative protein abundance in the validation cohort and phosphosite enrichment levels among clinical subgroups

A. Box plots of relative protein abundance in the validation cohort among clinical subgroups.

B. Box plots of phosphosite enrichment levels in the validation cohort among clinical subgroups.

Supplementary Figure 7: Box plots illustrating the metabolomic enrichment levels among clinical subgroups

Supplementary Figure 1

A

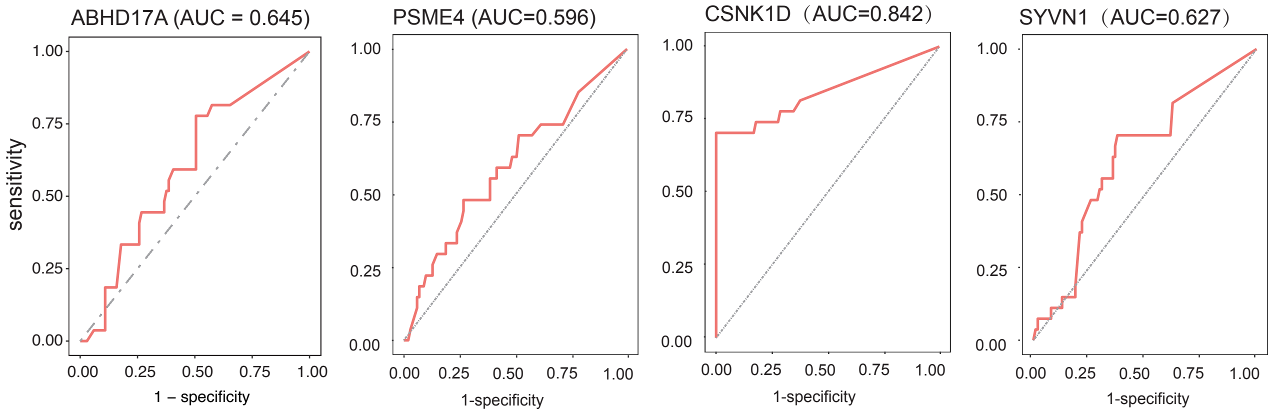

B

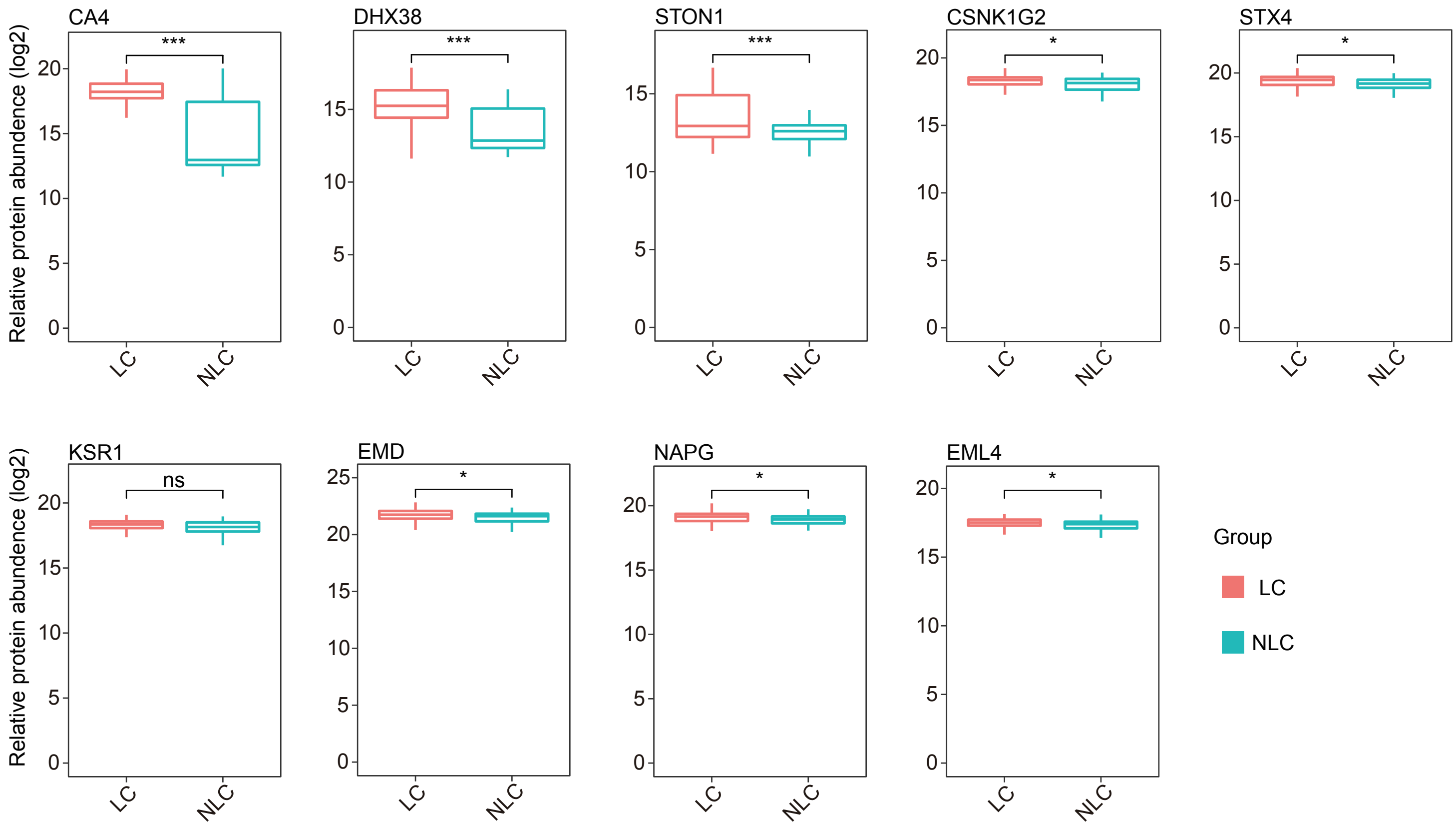

Supplementary Figure 2

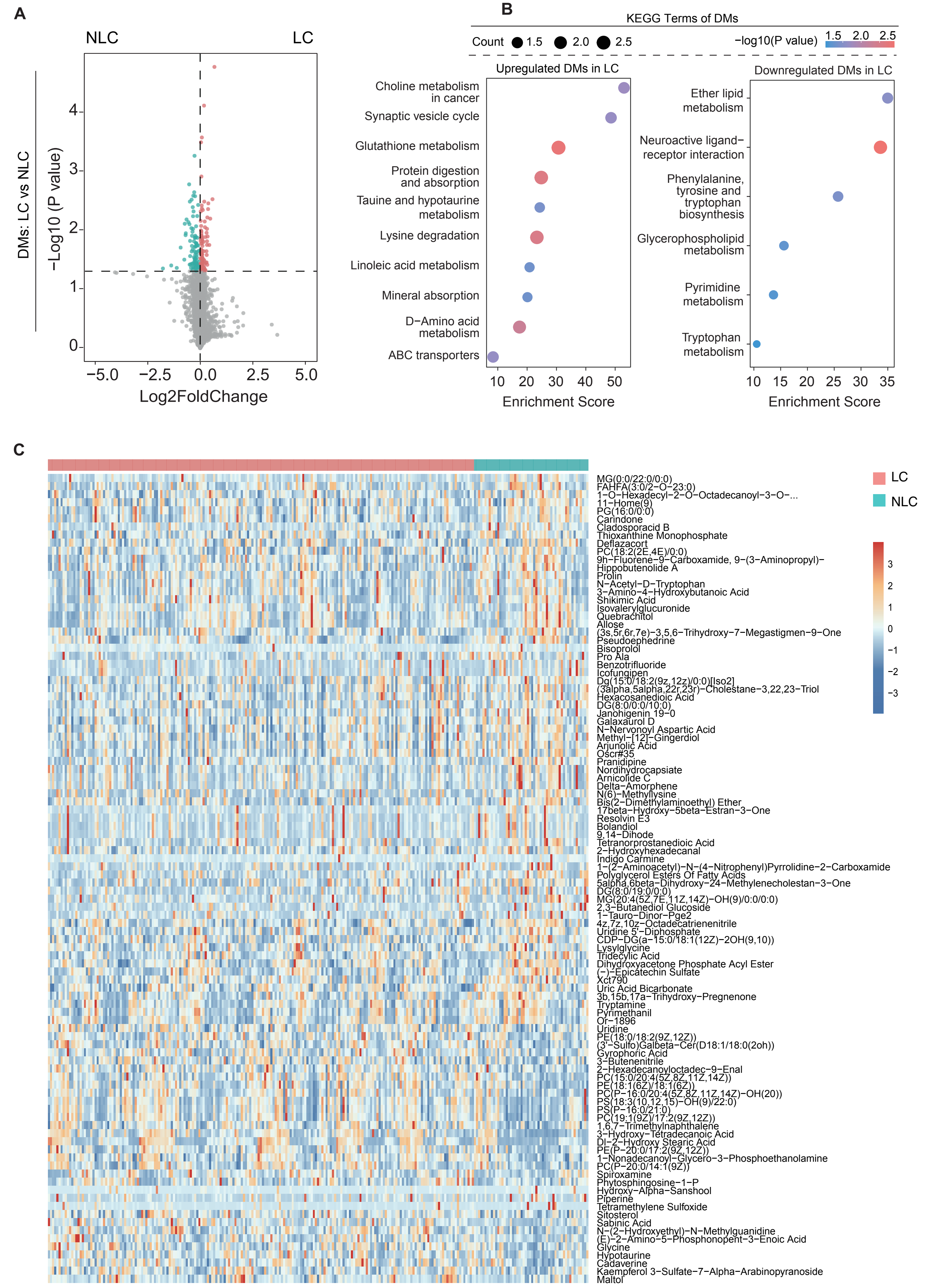

Supplementary Figure 3

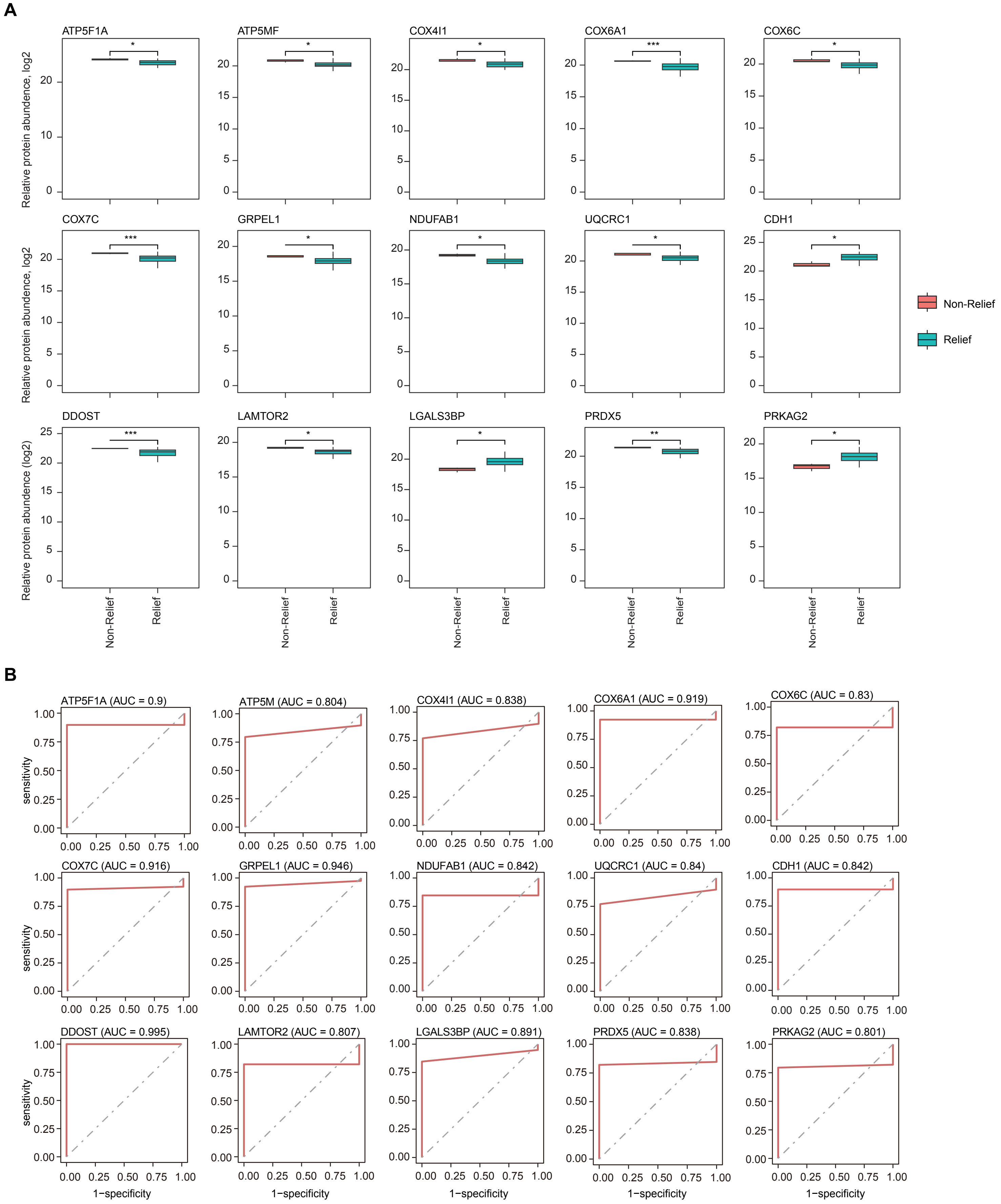

Supplementary Figure 4

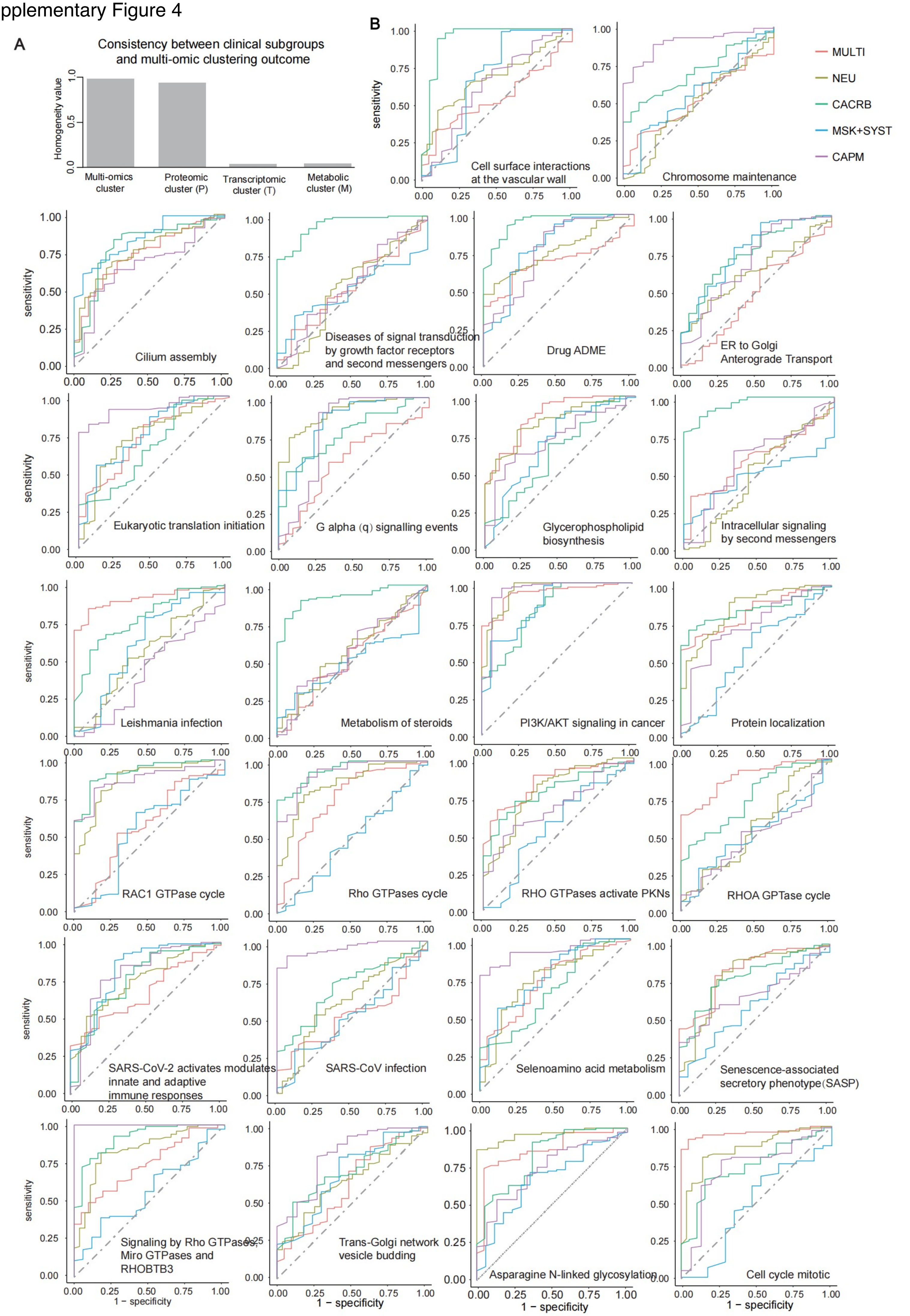

Supplementary Figure 5

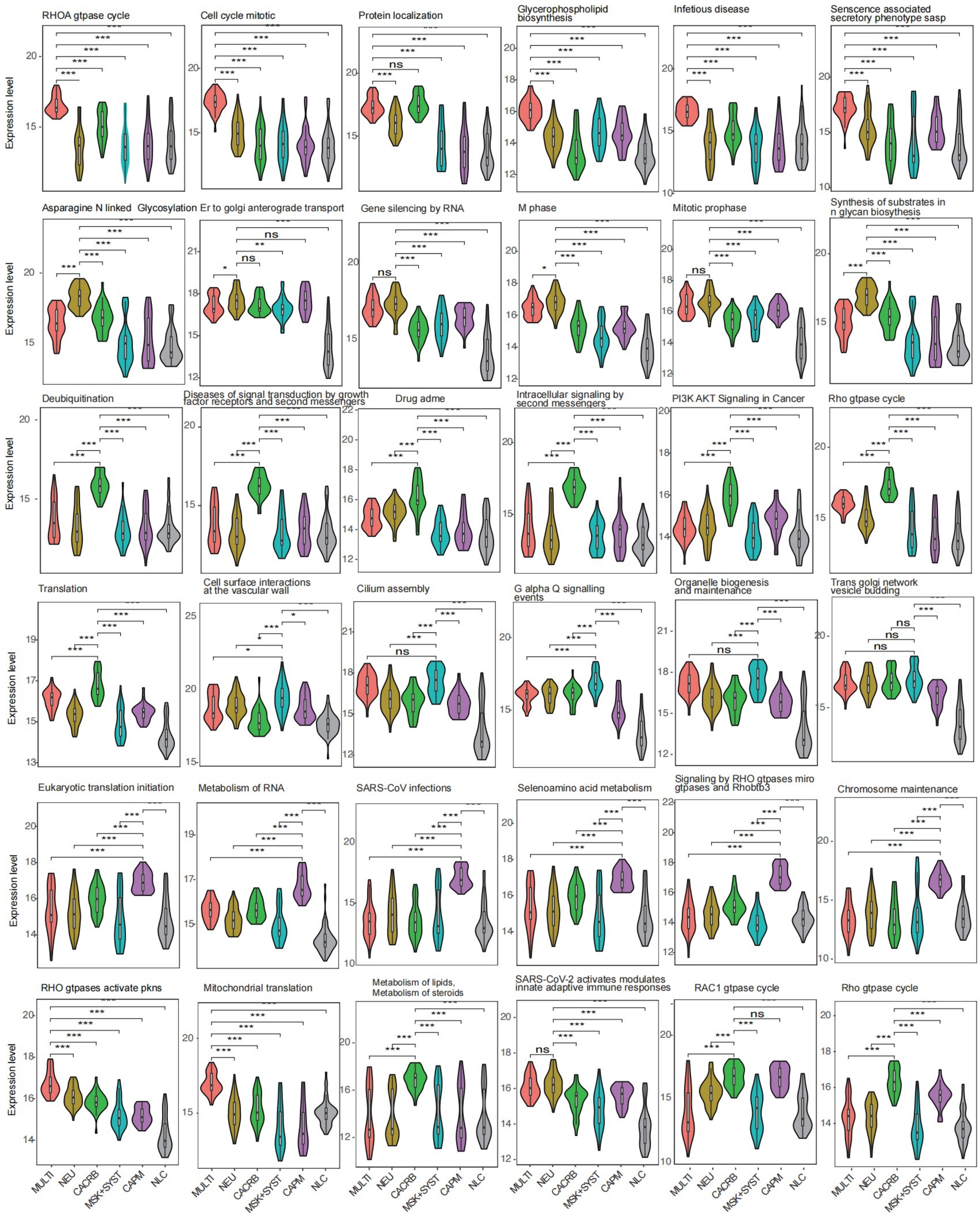

Supplementary Figure 6

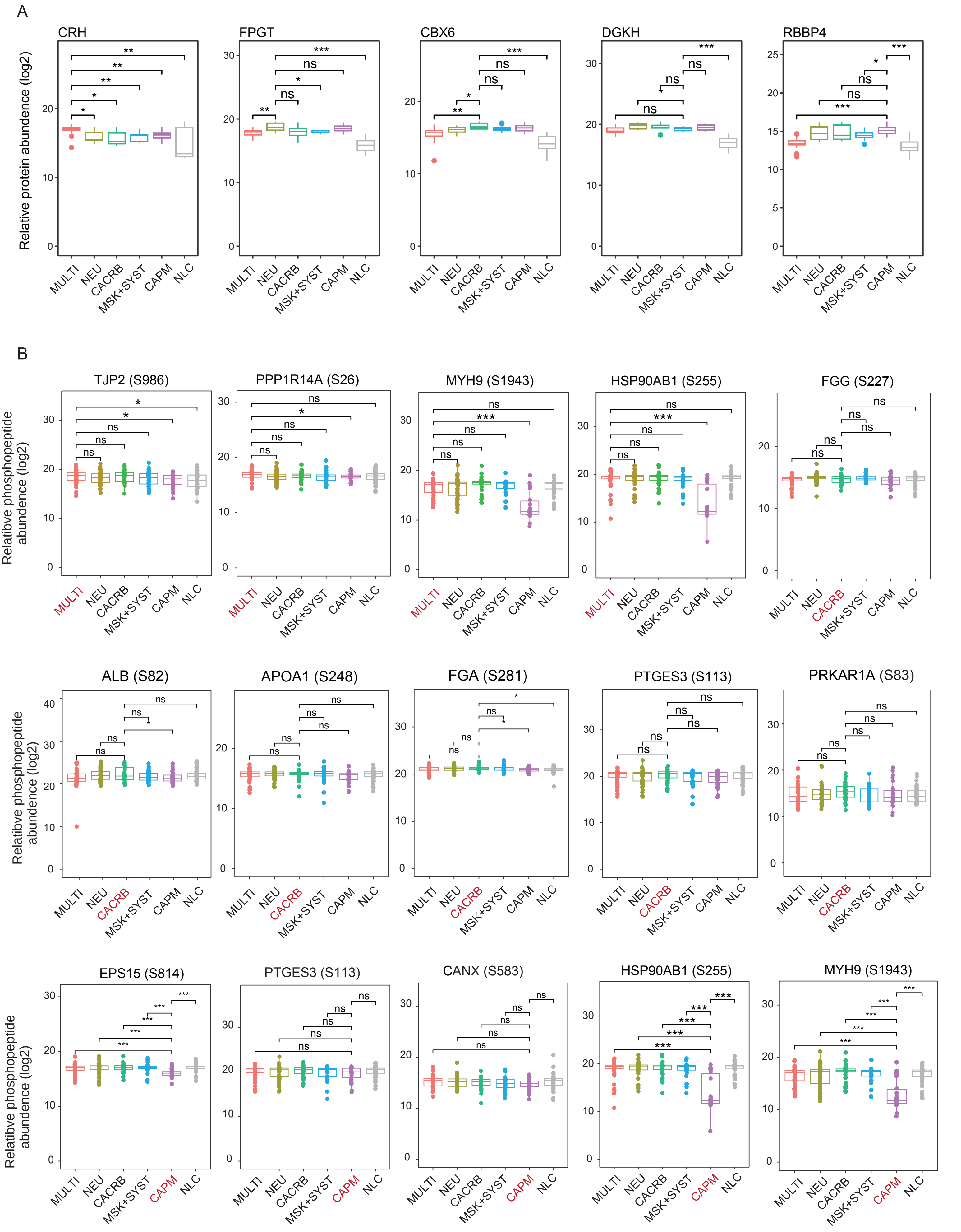

Supplementary Figure 7

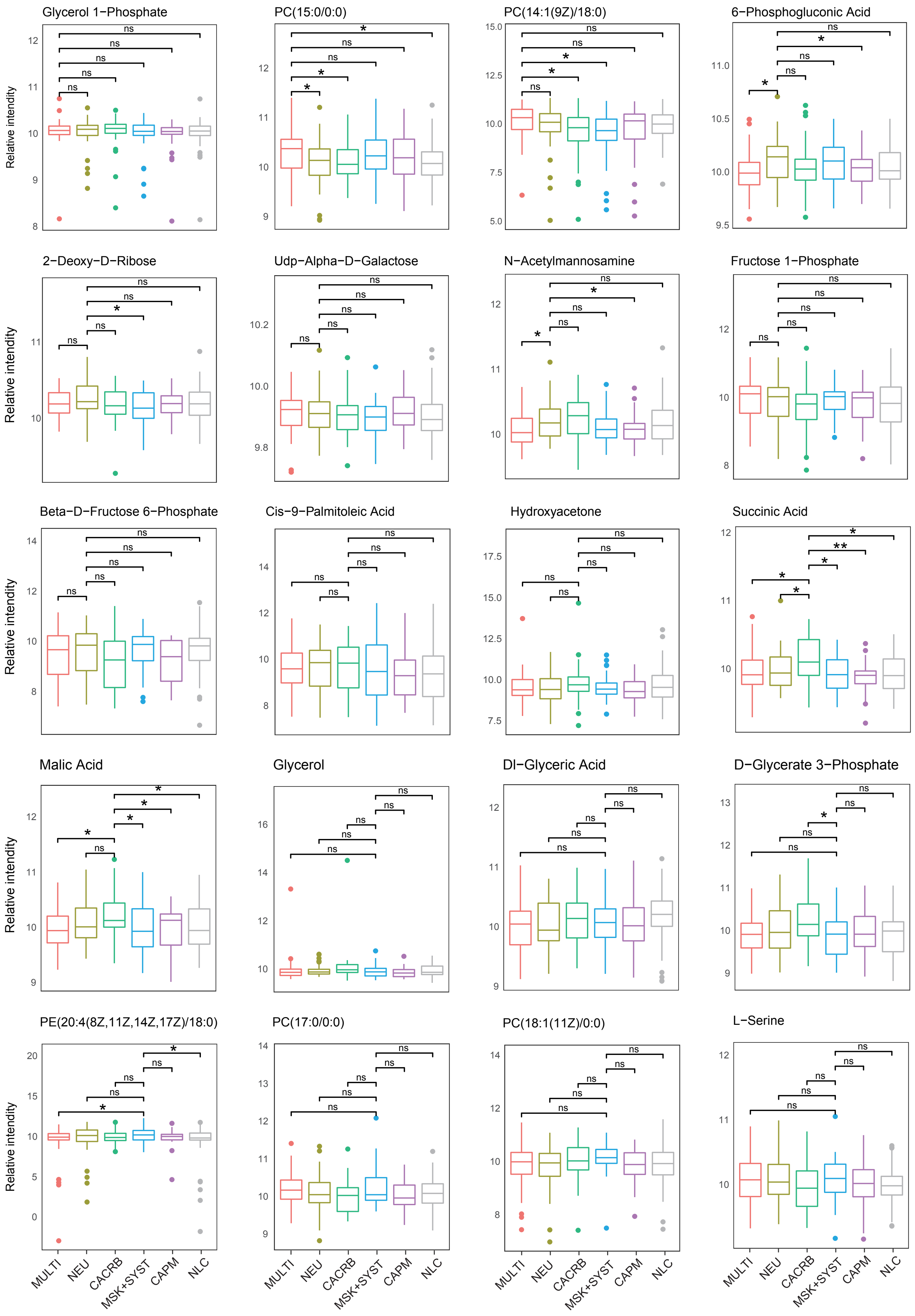

Supplementary Table 1 Imaging examination and clinical laboratory examinations of participants

	MULTI (n=43)	CAPM (n=30)	CACRB (n=37)	NEU (n=56)	MSK+SYST (n=35)	NLC (n=56)	*P* value
**Gender (Male)**	23/43(53.49)	17/30(56.67)	25/37(67.57)	36/56 (64.29)	15/35(42.86)	33/56(58.93)	0.3068704
**Chest CT**							
**Normal CT pattern**	15/42(35.71)	15/30(50.00)	12/36(33.33)	27/48(56.25)	14/33(42.42)	21/51(41.18)	0.27177575
**Ground-glass opacity**	0/42(0)	2/30(6.67)	0/36(0.00)	0/48(0.00)	1/33(3.03)	1/51(1.96)	0.15159364
**Old lesions**	15/42(35.71)	6/30(20.00)	13/36(36.11)	6/48(12.50)	11/33(33.33)	14/51(27.45)	0.08553647
**Chronic slight inflammation**	5/42(2.07)	4/30(13.33)	3/36(8.33)	4/48(8.33)	4/33(12.12)	5/51(9.80)	0.96611219
**Pleural thickening**	2/42(4.76)	0/30(0.00)	2/36(5.56)	4/48(8.33)	0/33(0.00)	2/51(3.92)	0.46818414
**Laboratory examinations**							
**Autoantibody**	10/43(23.26)	8/30(26.67)	7/37(18.92)	11/56(19.64)	8/35(22.86)	15/56(26.79)	0.92553475
**NT-Pro-BNP**	19.05(5-70)	19.43(5-67)	967.08(5-35000)	23.57(5-160)	18.17(5-76)	37.23(5-938)	0.31663781
**C-reactive protein**	2.98(1-32)	1.50(1-6)	2.08(1-14)	1.87(1-15)	1.37(1-4)	2.32(1-26)	0.333875
**D-Dimer**	0.2(0-0.7)	0.18(0-0.4)	0.25(0-0.9)	0.22(0-1.1)	0.23(0-0.8)	0.20(0-1.3)	0.5591215
**Procalcitonin**	0.03(0.02-0.06)	0.03(0.02-0.05)	0.05(0.02-0.28)	0.03(0.02-0.06)	0.03(0.02-0.04)	0.04(0.02-0.73)	0.27268836
**ESR**	9.16(1-25)	7.86(0-16)	8.36(2-40)	8.35(1-40)	8.57(2-28)	8.07(1-32)	0.95517373
**White blood cell count**	6.05(2.56-13.3)	6.17(3.97-8.67)	6.56(3.36-12.42)	6.18(3.12-10.51)	5.97(3.67-8.50)	6.33(4.01-10.41)	0.57694762
**Red blood cell count**	4.71(3.69-5.66)	4.84(4.16-5.96)	4.84(3.72-6.32)	4.75(3.68-6.02)	4.66(3.76-5.74)	4.86(3.69-6.03)	0.42300115
**Lymphocyte count**	1.81(0.94-2.6)	2.06(1.16-4.13)	2.00(1.06-3.24)	1.97(0.94-3.43)	1.81(0.88-3.93)	2.02(1.11-3.59)	0.19280494
**Monocyte count**	0.37(0.18-0.89)	0.36(0.17-0.6)	0.39(0.25-0.84)	0.36(0.15-0.73)	0.33(0.19-0.55)	0.39(0.16-0.77)	0.10018383
**Neutrophil count**	3.68(1.36-10.24)	3.59(1.66-6.22)	4.01(1.74-8.94)	3.70(1.74-7.35)	3.71(2.07-7.06)	3.78(1.82-7.5)	0.78593002
**Platelet**	259.86(134-373)	252.00(185-416)	249.31(122-458)	243.41(154-337)	252.09(159-306)	260.02(139-405)	0.61230845
**INR**	0.88(0.74-0.98)	0.86(0.74-1.01)	0.86(0.7-1.15)	0.89(0.7-1.1)	0.89(0.68-1.06)	0.90(0.74-1.75)	0.09996158
**APTT**	29.96(22-48.7)	29.50(21.2-52.5)	29.40(20.9-38.7)	31.73(21.7-46.7)	29.86(19.6-40.2)	28.97(19-41)	0.14642261
**Albumin**	48.45(41.1-54.2)	47.91(5-52.8)	49.16(37.6-55.2)	48.16(15.0-57.0)	49.78(44.2-57.0)	48.54(20-55.8)	0.69291034
**ALT**	21.91(3-63)	21.06(2.7-43)	34.86(10-139)	32.61(4-290)	18.74(4-47)	23.09(5-135)	0.06263425

**AST**	22.3(14-46)	22.07(13-81)	27.81(10-83)	23.12(11-61)	20.40(12-48)	22.53(11-72.9)	0.12987416
**Total bilirubin**	10.22(3-23.1)	13.36(4.9-91)	10.79(3.4-24)	14.06(3.3-96)	12.01(3.6-32.4)	12.05(4.4-79)	0.51829426
**Creatine kinase**	133.49(42-687)	129.20(47-664)	122.57(35-482)	113.11(22.6-335)	181.66(25-2942)	119.55(30-530)	0.69081231
**Serum creatine**	69.37(44-104)	74.23(53-108)	99.65(45-960)	69.14(16.3-100)	69.46(43-130)	70.86(46-118)	0.13501495
**Glomeruar filtration rate**	103.27(67-147)	95.44(3.2-131)	93.81(4-138)	101.73(11.5-167)	102.84(50-142)	103.69(11.6-149)	0.16963933
**Ferritin**	222.25(3.51-756.3)	185.55(5.15-645.7)	283.11(4.42-1066)	225.78(5.65-716.6)	161.27(6.74-631.3)	229.28(10.74-801.9)	0.09236631

ESR, erythrocyte sedimentation rate. INR, international normalized ratio. APTT, activated partial thromboplastin time. ALT, Alanine aminotransferase. AST, aspartate aminotransferase
