# Supplementary material for: Integrated multi-omics characterization across clinically relevant subgroups of long COVID
Source: Natl Sci Rev. 2024 Nov 15;12(8):nwae410. doi: 10.1093/nsr/nwae410 (PMC12365753; doi:10.1093/nsr/nwae410)
Supplement: nwae410_Supplemental_Files [file nwae410_supplemental_files.zip › Supplementary method.docx]

**Supplementary Material**

**Methods**

**Proteomic and Phosphoproteomics sample preparation and quantification analysis**

*Nanomagnetic bead-mediated enrichment of low-abundance plasma proteins*

Low abundance proteins in serum samples were enriched using the EasyPept DeeP kit for protein enrichment and pre-treatment (Omicron, Shanghai, China). Following the manufacturer’s protocol, 1 mg (40 µL) of magnetic nanoparticle suspension was utilized, and the magnetic beads were isolated via magnetic separation to remove the supernatant. After several washes, the magnetic beads were resuspended, and 100 µL of serum was added before incubation at 37°C using a flip mixer for 1 hour with 360° rotation. Subsequently, the supernatant was separated using magnetic separation, followed by the addition of 300 µL of washing solution and gentle shaking for 5 minutes. This washing step was repeated thrice. The proteins were enzymatically hydrolyzed to generate peptides, followed by reductive alkylation and desalting. The total peptide concentration was determined using the nanodroplet method.

*Data independent acquisition (DIA)Mass spectrometry analysis*

The Proteomic data analysis was performed by Shanghai Luming biological technology co., LTD (Shanghai, China). All analyses were performed by a TimsTOF Pro mass spectrometer (Bruker) equipped with an EASY-nLCTM 1200 system (Bruker). The gradient settings of EASY-nLCTM 1200 system were as follows: 0-45 min, 5-27% B; 45-50 min, 27-46% B; 50-55 min, 46-100% B; 55-60 min, 100% B. The mass spectrometry conditions were set as: capillary voltage of 1.4 kV, drying gas temperature of 180°C, drying gas flow rate of 3.0 L/min, mass scan range from 100 to 1700 m/z, ion mobility range between 0.7 and 1.3 Vs/cm², and collision energy range of 20-59 eV.

*Phosphorylated peptide enrichment*

Using IMAC to enrich phosphorylated peptides. The lyophilized peptide was first suspended in 200uL binding/WashBuffer. The second step was to balance the columns. The suspension peptides of 200uL are then added to the equilibrated column, incubated for 30 min. Then place the column in a centrifuge tube, centrifuging the tube at 1000g for 30 sec. Place the column into a new centrifuge tube, add 100uL of elution buffer, centrifuge at 1,000g for 30 sec, repeat once. Dry the elution buffer in a high-speed vacuum concentrator.

All analyses were performed by a timsTOF Pro mass spectrometer (Thermo, Bruker) equipped with an Easyspray source (Thermo, USA). Nanoflow reversed-phase chromatography was performed on an EASY-nLC 1200 system (Thermo Fisher Scientific). Mobile phases A and B were 0.1 vol% formic acid solution and 80: 20:0.1 vol% ACN: water: formic acid, respectively. The total run was 60 min (0~45 min, 5-27% B; 45~50 min, 27-46% B; 50~55 min, 46-100% B；55~60 min, 100% B). Capillary voltage was 1.4 kV, dry gas temperature was 180 ℃, and dry gas flow rate was 3.0 L/min. The full MS scan range was set from 100 to 1700 m/z. The ion mobility range was 0.7-1.3 vs/cm2, and the collision energy range was 20-59 ev.

*Database search for Proteomic data*

The default factory settings were used for the Spectronaut Pulsar 18.4 (Biognosys, Swiss) search and library generation (including Trypsyin/P as enzyme, up to two missed cleavages allowed Oxidation of Me as variable modifications, carbamidomethyl as fixed modification, and 1% FDR for PSM, peptide and protein identification). The DDA search results were imported into Spectronaut Pulsar™. The DIA data were analyzed with Spectronaut searching the above constructed spectral library. Main parameters of the software were set as follows: Precursor Qvalue cutoff and Protein Qvalue cutoff were set as 0.01, Normalization Strategy was set as Local Normalization, and use MS2 as Quantity MS-Level.

*Database search for Phosphoproteomics data*

MS/MS spectra were searched using the Spectronaut Pulsar™ 18.4 (Biognosys, Swiss) against the Uniprot Homo sapiens (v9606-2023.2.1) database. Search database specific parameters are set as follows: Fixed modifications：Carbamidomethyl(C) ; Variable modification：phospho (STY), Oxidation (M) and Acetyl (Protein N-term); digestion: trypsin; Precursor Qvalue cutoff: 0.01; Protein Qvalue cutoff: 0.01; Missed cleavage: 2; Quantity MS-Level: MS2.

*Quantification and quality control analysis*

Average CVs for the iRT value of 11 peptides is 1.53%. To ensure the stability of the liquid quality system and the reliability of qualitative and quantitative data, a QC sample (an equal mix of all samples) should be inserted into the sample queue at predefined intervals. The mixed-QC samples should be monitored during the on-machine process to guarantee system stability. Qualitative analysis of all mixed-QC data showed the average overlap count is 94.47%, with the aim of achieving an overlap count of at least 80%. At last, a total of 3305 proteins expressed were identified as belonging to the proteome of serum in this study. And a total of 392 phosphorylated peptides were identified as belonging to 190 proteins in this study. Raw data was retrieved through database search, and proteins with Unique Peptides >= 1, samples with valid values >= 2, and proteins in groups with valid values representing at least 50% of the total were retained. Missing values in groups with >= 50% valid values were imputed using the group mean, while the remaining empty values were filled with half of the minimum sample value. All data was then subjected to median normalization and log2 transformation to ensure the reliability of the identified proteins.

**RNA extraction and RNA-seq**

*RNA Isolation and Library Preparation*

Total RNA was extracted using the TRIzol reagent (Invitrogen, CA, USA) according to the manufacturer’s protocol. RNA purity and quantification were evaluated using the NanoDrop 2000 spectrophotometer (Thermo Scientific, USA). RNA integrity was assessed using the Agilent 2100 Bioanalyzer (Agilent Technologies, Santa Clara, CA, USA). Then the libraries were constructed using VAHTS Universal V6 RNA-seq Library Prep Kit according to the manufacturer’s instructions. The transcriptome sequencing and analysis were conducted by OE Biotech Co., Ltd. (Shanghai, China).

**Metabolomic Sample Preparation and quantification analysis**

*Sample Preparation*

Samples stored at -80℃ were thawed at room temperature. 150μL of sample was added to a 1.5 mL Eppendorf tube with 600μL methanol-acetonitrile mixture (V: V=2:1, containing 4μg/mL L-2-chlorophenylalanine as internal standard). Subsequently, the whole samples were extracted by ultrasonic for 10 min in ice-water bath, stored at -20℃ for 30 min. The extract was centrifuged at 4°C (12000 rpm) for 10 min. 150μL of supernatant was filtered by 0.22μm and stored at -80 ℃for LC-MS/MS analysis. Another 150μL of supernatant was dried in a freeze concentration centrifugal dryer. 80μL of 15mg/mL methoxylamine hydrochloride in pyridine was subsequently added. The resultant mixture was vortexed vigorously for 2 min and incubated at 37°C for 60 min. 50μL of BSTFA (with 1% TMCS) and 10μL internal standards mixture (C8/C9/C10/C12/C14/C16/C18/C20/C22/C24, solved in chloroform) were added into the mixture, which was vortexed vigorously for 2 min and then derivatized at 70°C for 60 min. The samples were placed at ambient temperature for 30 min before GC-MS analysis. QC samples were prepared by mixing aliquot of all samples to be a pooled sample.

*GC-MS analysis*

The metabolomic data analysis was performed by Shanghai Luming biological technology co., LTD (Shanghai, China). The derivatived samples were analyzed on an Agilent 7890B gas chromatography system coupled to an Agilent 5977A MSD system (Agilent Technologies Inc., CA, USA). A DB-5MS fused-silica Capillary Column (30m × 0.25mm × 0.25μm, Agilent J & W Scientific, Folsom, CA, USA) was utilized to separate the derivatives. Helium (> 99.999%) was used as the carrier gas at a constant flow rate of 1 mL/min. The injector temperature was maintained at 260℃. Injection volume was 1μL by splitless mode. The initial oven temperature was 60℃ held at 60 °C for 0.5 min, ramped to 125℃ at a rate of 8℃/min, to 210℃ at a rate of 8℃/min, to 270℃ at a rate of 15℃/min, to 305℃ at a rate of 20℃/min, and finally held at 305℃ for 5min. The temperature of MS quadrupole and ion source (electron impact) was set to 150 and 230℃, respectively. The collision energy was 70 eV. Mass spectrometric data was acquired in a full-scan mode (m/z 50-500), and the solvent delay time was set to 5 min.

*LC-MS/MS analysis*

The metabolomic data analysis was performed by Shanghai Luming biological technology co., LTD (Shanghai, China). An ACQUITY UPLC I-Class plus (Waters Corporation, Milford, USA) fitted with Q-Exactive mass spectrometer equipped with heated electrospray ionization (ESI) source (Thermo Fisher Scientific, Waltham, MA, USA) was used to analyze the metabolic profiling in both ESI positive and ESI negative ion modes. An ACQUITY UPLC HSS T3 column (1.8μm, 2.1 × 100 mm) were employed in both positive and negative modes. The binary gradient elution system consisted of (A) water (containing 0.1 % formic acid, v/v) and (B) acetonitrile and separation was achieved using the following gradient: 0-2 min, 5% B; 4min, 30% B; 8min, 50% B; 10min, 80% B; 14-15 min, 100% B; 15.1 min, 5% and 16 min, 5%B. The flow rate was 0.35 mL/min and column temperature were 45℃. All the samples were kept at 10℃ during the analysis. The injection volume was 3μL.

The mass range was from m/z 100 to 1,000. The resolution was set at 70,000 for the full MS scans and 17500 for HCD MS/MS scans. The collision energy was set at 10, 20 and 40 eV. The mass spectrometer operated as follows: spray voltage, 3800 V (+) and -3000 V (−); sheath gas flow rate, 35 arbitrary units; auxiliary gas flow rate, 8 arbitrary units; capillary temperature, 320°C; Aux gas heater temperature, 350°C; S-lens RF level, 50.

*Data Preprocessing and Statistical Analysis*

The obtained GC/MS raw data in. D formats were transferred to .abf format via software Analysis Base File Converter for quick retrieval of data. Then, data were imported into software MS-DIAL, which performs peak detection, peak identification, MS2Dec deconvolution, characterization, peak alignment, wave filtering, and missing value interpolation. Metabolite characterization was based on a self-built LUG database. A data matrix was derived. The three-dimensional matrix includes sample information, the name of the peak of each substance, retention time, retention index, mass-to-charge ratio, and signal intensity. In each sample, all peak signal intensities were segmented and normalized according to the internal standards with RSD < 0.1 after screening. After the data was normalized, redundancy removal and peak merging were conducted to obtain the data matrix.

As for LC-MS/MS data, the original data were processed by software Progenesis QI V3.0 (Nonlinear, Dynamics, Newcastle, UK) for baseline filtering, peak identification, integral, retention time correction, peak alignment, and normalization. Main parameters of 5 ppm precursor tolerance, 10 ppm product tolerance, and 5% product ion threshold were applied. Compound identifications were based on precise mass-to-charge ratio (M/z), secondary fragments, and isotopic distribution using The Human Metabolome Database (HMDB), Lipidmaps (V2.3), Metlin, and self-built databases. The extracted data were then further processed by removing any peaks with a missing value (ion intensity = 0) in more than 50% in groups, by replacing zero value by half of the minimum value, and by screening according to the qualitative results of the compound. Compounds with resulting scores below 36 (out of 60) points were also deemed to be inaccurate and removed. A data matrix was combined from the positive and negative ion data. All data was then subjected to median normalization and log2 transformation to ensure the reliability of the identified markers.

**Single cell collection, sorting, library preparation and sequencing**

PBMCs were isolated using HISTOPAQUE-1077 (Sigma-Aldrich, 10771) solution according to the manufacturer’s instructions. Briefly, 4 mL of fresh peripheral blood was collected in EDTA anticoagulant tubes and subsequently layered onto HISTOPAQUE-1077. After centrifugation, PBMCs remained at the plasma-HISTOPAQUE-1077 interface and were carefully transferred to a new tube. Erythrocytes were removed using red blood cell lysis buffer and washed twice with sorting buffer (PBS supplemented with 2% fetal bovine serum). The cell pellets were re-suspended in sorting buffer and were subsequently passed through a 40 µm Flowmi Cell Strainer. Thaw frozen PBMC cells according to the User Guide CG00039_Demonstrated Protocol Fresh Frozen Human PBMCs. Briefly, the frozen PBMC cells were thawed in the water bath at 37°C and washed in warm complete growth medium (RPMI medium with 10% fetal bovine serum). The cell pellets were re-suspended in sorting buffer and were subsequently passed through a 40 µm Flowmi Cell Strainer. Single cell suspensions were stained with 7AAD for FACS sorting, performed on a BD Melody instrument. Determine cell viability and cell concentration using a Countstar Automated Cell Counter. Cell viability of PBMCs were greater than 90% and the cell concentration was adjusted to 500-1200 cells/uL. PBMCs were loaded 18,000 cells/chip position using the 10x Chromium Next GEM Single Cell 5' Kit v2. All the subsequent steps were performed following the standard manufacturer’s protocols. Purified libraries were analyzed by an Illumina nova-seq 6000 sequencer with 150-bp paired-end reads.

**Multi-omics data analysis**

*RNA Sequencing analysis*

Quality control. The first step in our data analysis was quality control to ensure that raw data had good metrics and no significant biases which may affect the following analysis. In this study, RseqQC (v 4.0.0) with parameters was applied to calculate read quality for all samples.

Filter of raw data. The adapter sequence of raw data (Fastq) was removed using fastp (v0.20.1). Then, the reads with more than 50 bp were preserved (named clean reads) and used for downstream analysis.

Sequence alignment. Then the clean reads were mapped to the human (Homo sapiens) genome (GRCh38.p13) by using hisat2 (v2.1.0) software with default parameters and were annotated with transcriptome database (GENCODE v19).

Gene expression estimation. Expression estimation of gene and transcript was performed by using htseq-count (v0.12.3) with -s reverse parameter with other parameters in default. The relative abundance of the transcript was quantified based on normalized metric named FPKM.

*Proteomics data analysis*

For the proteome data, limma R package was implemented and moderated t-statistics was used. We selected the trend parameter to enhance the stability of the analysis and genes with FDR <0.05 and fold change >1.5 or other thresholds were considered as significantly changed genes. These significant genes were then performed Reactome Pathway enrichment analysis using clusterProfiler R package and an FDR value <0.05 or other thresholds were considered as the cutoffs of significantly regulated pathways.

*Metabolomics data analysis*

For the metabolism data, the matrix was imported in R to carry out Principle Component Analysis (PCA) to observe the overall distribution among the samples and the stability of the whole analysis process. Orthogonal Partial Least-Squares-Discriminant Analysis (OPLS-DA) and Partial Least-Squares-Discriminant Analysis (PLS-DA) were utilized to distinguish the metabolites that differ between groups. To prevent overfitting, 7-fold cross-validation and 200 Response Permutation Testing (RPT) were used to evaluate the quality of the model. Variable Importance of Projection (VIP) values obtained from the OPLS-DA model were used to rank the overall contribution of each variable to group discrimination. A two-tailed Student’s T-test was further used to verify whether the metabolites of difference between groups were significant. Differential metabolites were selected with VIP values greater than 1.0 and P-values less than 0.05.

**Multi-omics clustering analysis for transcriptome, proteome and metabolome.**

We performed multi-omics clustering for mRNA, protein and metabolite abundance using similarity network fusion (SNF) in 255 patients with overlapped datasets. Multi-omics clustering was performed to identify subclusters of long COVID patients using the similarity network fusion (SNF) in 255 patients with overlapped datasets. We selected the proteins from the proteins expressed in at least 50% of the samples and cv>0.1; genes from RNA-seq data with cv>1 and metabolomes from metabolomics enrichment data with cv>1 for clustering across 255 samples. The consensus matrices of k = 6, 7, 8, 9 and 10 clusters. We then determined the consensus matrix of k=6 as the best solution for clustering as it presents the most separated clusters (Supplementary table 2).

**Functional enrichment analysis**

To further elucidate the biological characteristics of the clinical subgrouping, GSEA analysis was performed to identify the pathway alterations among the 6 subgroups using function GSEA in clusterProfiler (v4.2.2) package. Upregulated genes and proteins of each group were selected to achieve enrichment scores over REACTOME database of MsigDB database v.7.1 with at least 2 overlapping genes.

In each subgroup, the normalized enrichment scores (NES) of terms from RNA-seq and protein levels should both higher than 0. We identified 51 pathways using the NES with the cutoff of 1, and 28 distinct pathways through knowledge-based selection were visualized by heatmap.

**Potential prognostic protein biomarker detection**

To identify potential protein biomarkers, we applied MNN models to identify the diagnose marker combination for clinical subtypes. Patients divided into two partitions used to train and validate the MNN models. That is, the MNN models were trained on the 70% of patients sampled randomly and validated on those from the remaining partition (30% of all patients). We then measured the accuracy of each marker using ROC analysis. We selected the AUC>0.8 as biomarker candidates and the potential best prognostic biomarkers combination were identified with the highest sum of AUC values.
